# Epidural analgesia is not superior to systemic postoperative analgesia with regard to preventing chronic or neuropathic pain after thoracotomy

**DOI:** 10.1186/1749-8090-8-127

**Published:** 2013-05-13

**Authors:** Sandra Kampe, Joachim Lohmer, Gerhard Weinreich, Moritz Hahn, Georgios Stamatis, Stefan Welter

**Affiliations:** 1 Department of Anaesthesiology and Pain Medicine, Ruhrlandklinik, West German Lung Center University Hospital Essen, Tüschener Weg 40, Essen 45239, Germany; 2 Department of Anaesthesiology and Intensive Care Medicine, Hospital Cologne-Merheim, University of Witten-Herdecke, Witten, Germany; 3 Department of Pneumology, Ruhrlandklinik, West German Lung Center, University Hospital Essen, Essen, Germany; 4 Institute of Medical Statistics, Informatics and Epidemiology, University of Cologne, Cologne, Germany; 5 Department of Thoracic Surgery and Thoracic Endoscopy, Ruhrlandklinik, West German Lung Center, University Hospital Essen, Essen, Germany

## Abstract

**Background:**

To assess prospectively the incidence of chronic and neuropathic pain in patients undergoing anteroaxillary thoracotomy with postoperative epidural analgesia or controlled-release oxycodone pain regimen.

**Methods:**

77 patients who underwent anteroaxillary thoracotomy were enrolled in our observational study. 40 patients received postoperatively a standardized oral analgesic protocol with controlled-release oxycodone and IV non opioid (CRO Group), and 37 patients received epidural analgesia with ropivacaine 0.1% + 1 μg/ml sufentanil (EDA Group) and IV non opioid. The painDETECT questionnaire was completed from the patients with one of the authors (JL) on the 7th postoperative day and six months postoperatively.

**Results:**

The data of 60 patients were eligible for statistical analysis, 28 patients in the CRO Group and 32 patients in the EDA Group. 17 patients did not reach the 6-months follow-up interval (12 drop outs in the CRO Group and 5 drop outs in the EDA Group). 79% percent of patients in the CRO Group and 74% percent of patients in the EDA Group had a numeric rating scale score (NRS) = 0 after 6 months. 22% percent of patients in the CRO Group and 16% percent of patients in the EDA Group experienced a NRS 1–3 6-months postoperatively. No patient in the CRO Group and 9% percent of patients in the EDA Group had 6-months postoperatively a NRS 4–6. Neither in the CRO Group nor in the EDA Group we could detect a neuropathic pain 6 months postoperatively corresponding to a painDETECT score > 18. Overall, with regard to NRS, there was no statistical difference between the two groups (p = 0.13). 90% percent of patients in the CRO Group and 90% percent of patients in the EDA Group showed 6-months postoperatively a painDETECT score < 13 (definitely no neuropathic pain), and 9% percent in the EDA Group and 11% in the CRO Group had a 6-months painDETECt score 13–18 (p = not significant).

**Conclusion:**

These pilot data indicate that epidural analgesia is not superior to systemic postoperative analgesia with regard to preventing chronic or neuropathic pain after thoracotomy.

## Background

Pain after thoracic surgery is supposed to be one of the most recognized pain syndrome associated with a specific procedure [1]. Reports indicated that 50% of patients describe a chronic postthoracotomy pain (CPP) one year after the procedure [2], more recent data suggest that CPP might appear in 21% of patients one year after surgery when perioperative pain is managed aggressively [1].

To date regional anaesthesia is recommended for postoperative analgesia after thoracotomy [1,3,4]. Therefore published data on the incidence of CPP are commonly based on postoperative analgesia with regional techniques [5-7]. To our knowledge to date there are no prospective data available with regard on the incidence of CPP and neuropathic pain after thoracotomy with epidural analgesia (EDA) compared to a systemic pain regimen with controlled-release oxycodone (CRO).

The objective of the present study was to assess prospectively the incidence of chronic and neuropathic pain in patients undergoing anteroaxillary thoracotomy with postoperative epidural analgesia or controlled-release oxycodone pain regimen over a 6-months period.

## Methods

After obtaining local research committee approval (Ethics Committee of the University of Essen-Duisburg) and written informed consent, 77 patients were enrolled in our prospective observational study, conducted according to the Declaration of Helsinki.

Eligible patients were those scheduled for thoracotomy, aged 18–70 years and American Society of Anesthesiologists (ASA) physical status I-IV. Patients were not randomly assigned to one or the other observation group, the determination of the postoperative analgesic regimen was due to the discretion of the anaesthetist performing the preoperative interview with the patient. One of the authors (JL) included the patients into the study on the 2nd postoperative day. Patients with a preoperative pain history (numeric rating scale score NRS > 0) were not included. Further exclusion criteria were communication difficulties, psychiatric disease, pregnancy and a history of drug or alcohol abuse.

All patients underwent anteroaxillary thoracotomy in balanced anaesthesia. If an epidural catheter was inserted, it was placed before induction of anaesthesia and commenced after start of surgery continuously at 4–6 ml/h.

Half an hour before the end of the surgical procedure all patients received 2.5 g of IV dipyrone. Dipyrone is a non-opioid analgesic frequently used in the perioperative setting in Germany. After extubating the trachea patients received IV piritramide at the discretion of the anaesthetist. Piritramide is an opioid commonly used in Germany with approximately 0.7 times the potency of morphine. Patients were admitted to the intensive care unit for one night and received 1 g of dipyrone every 6 hours and IV piritramide bolus doses when NRS (numeric rating scale, 0 = no pain up to 10 = worst pain imaginable) was > 3. If patients had epidurals, the EDA was continued.

After discharge from the intensive care unit on the first postoperative day (POD) the systemic pain therapy patients received controlled-release oxycodone (CRO Group) 30–0 – 20 mg, and from the 2nd POD 20 – 0–20 mg and IV dipyrone 1 g every 6 h. If the NRS was > 4 IV rescue medication with IV paracetamol was administered (up to 15 mg/kg). The EDA Group received postoperatively epidural analgesia with ropivacaine 0.1% + 1 μg/ml sufentanil (EDA Group) and IV dipyrone 1 g every 6 h. On the fourth POD IV dipyrone 1 g was oralized.

All patients received anteroaxillary thoracotomy via the 4th intercostal space. The serratus muscle was not cut, but spread. At the end of surgery two 24 CH soft chest tubes were placed and the thoracic cavum was closed with three pericostal sutures.

During their stay in hospital all patients were visited daily by an anaesthetist specialized in pain therapy and pain medication was adjusted to the state of recovery. Moreover NRS at rest and on coughing were documented by the nurses on the wards every 6 h, implicating that the rescue medication had to be administered by the nurse when NRS scores > 3 at rest and > 5 on coughing. Thirty min after administration of rescue medication the NRS had to be re-assessed. If pain score was again > 5 on coughing, the nurse had to contact the anaesthetist responsible for postoperative pain therapy.

The painDETECT questionnaire was completed from the patients with one of the authors (JL) twice during the study period: on the 7th POD and 6-months postoperatively.

The painDETECT questionnaire was originally designed from the German Research network on Neuropathic Pain (Deutscher Forschungsverbund Neuropathischer Schmerz, DFNS) to find out how neuropathic pain symptoms are perceived by the patients. As a result, 7 questions that address the quality of neuropathic pain symptoms were included in the questionnaire (Table 1). Patients` answers were to be multiplicated with different multiplication factors generating a total score between 0–38. A total score from 0–12 assesses a neuropathic component to be very unlikely, a total score of 13–18 implicates that a neuropathic component might be possible, and a score between 19–38 confirms a neuropathic component.

**Table 1:** PainDETECT questionnaire

**(Item)**	**Score**
*Gradation of pain*	0
Do you suffer from a burning sensation (e.g. stinging ncttksj in the marked areas?	
• Do you have a tingling or prickling sensation in the area of your pain (like crawling ants or electrical tingling)?	0-5
• Is hght touching (clothing, a blanket) in this area painful?	0-5
• Do you have sudden pain attacks in the area of your pain, like electric chcks	0-5
• Is cold or heat (bath water) in this area occasionally painful?	0-5
• Do you suffer trom a sensation of numbness in the areas that you marked?	0-5
• Does slight pressure in this area, e.g. with a finger, trigger pain?	0-5
*Pain course pattern*	
Please select the picture that best describes the course of your pain:	
Persistent pain with slight fluctuations	0
Persistent pain with pain attacks	-1
Pain attacks without pain between them	+1
Pain attacks with pain between them	+1
*Radiating pain*	
Does your pain radiate to other regions of your body? Yes/No	+2/0

For each question: never, 0; hardly noticed. I slightly. 2; moderately. 3: strongly, 4; very strongly, 5 Questions used to document pain, but which were not used in the scoring, are not shown.

Cited from CMRO 2008;22 [10]:1911–20.

All variables were analyzed by methods of descriptive statistics (frequency, mean ± standard deviation, range). Differences between groups were analyzed by using the Mann–Whitney-U test and differences in proportions were statistically evaluated by using chi^2^ test. Statistical significance was determined at the p < 0.05 level.

Statistical analysis was performed by using SPSS 20.0 statistical package.

## Results

In 2008–2010,77 patients were enrolled in our study. The data of 60 patients were eligible for statistical analysis, 28 patients in the CRO Group and 32 patients in the EDA Group. 17 patients did not reach the 6-months follow-up interval (12 drop outs in the CRO Group and 5 drop outs in the EDA Group). The time of the surgical procedure was 147 ± 45 min in the CRO Group and 173 ± 59 min in the EDA Group (p = 0.08). The surgical procedures are presented in Table 2. In the CRO Group there were 9 females, in the EDA Group 5 female patients. 11 patients in the CRO Group were ASA II and 17 patients ASA III classified, in the EDA Group 6 patients were ASA II and 26 ASA III classified (p = 0.05). Results with regard to the NRS score 6-months postoperatively are presented in Table 3. 79% percent of patients in the CRO Group and 74% percent of patients in the EDA Group had a numeric rating scale score (NRS) = 0 after 6 months. 22% percent of patients in the CRO Group and 16% percent of patients in the EDA Group experienced a NRS 1–3 6-months postoperatively. No patient in the CRO Group and 9% percent of patients in the EDA Group had 6-months postoperatively a NRS 4–6. Overall, with regard to NRS, there was no statistical difference between the two groups (p = 0.13). The painDETECT scores 7-days and 3-months postoperatively are presented in Table 4. Neither in the CRO Group nor in the EDA Group we could detect a definite neuropathic pain 6 months postoperatively corresponding to a painDETECT score > 18. 90% percent of patients in the CRO Group and 90% percent of patients in the EDA Group showed 6-months postoperatively a painDETECT score < 13 (definitely no neuropathic pain), and 9% percent in the EDA Group and 11% in the CRO Group had a 6-months painDETECt score 13–18 (p = not significant).

**Table 2:** Surgical procedures

**Surgical procedures**	**CRO Group**	**EDA Group**
	**n = 28**	**n = 32**
Lobectomy	20	20
Pneumonectomy	3	6
Wedge resection	1	1
Segmentectomy	4	4
Others		1

*N*, Number of patients; *EDA*, Epidural Group; *CRO*, Controlled-release oxycodone Group.

**Table 3:** Percentages of patients with their NRS scores 6-months postoperatively in both groups

**NRS**	**CRO Group**	**EDA Group**	**p**
	** n = 28**	** n = 32**	
NRS 0	79%	74%	n.s.
NRS 1-3	22%	16%	n.s.
NRS 4-6	0%	9%	n.s.
NRS 7-10	0%	0%	n.s.

*CRO Group*, Systemic pain therapy group; *EDA Group*, Epidural analgesia group; *n*, Number of patients, NRS (numeric rating scale from 0 = no pain to 10 = worst pain imaginable) preoperatively, 6-months postoperatively.

**Table 4:** Percentages of patients with their painDETCET score 7-days postoperatively and 6-months postoperatively

**PainDETECT score**	**7-days postop.**		**6-months postop.**	
	**n**		**n**	
	**EDA**	**CRO**	**EDA**	**CRO**
0-12	90%	83%	90%	90%
13-18	3%	14%	9%	11%
> 19	3%	0%	0%	0%

*N*, Number of patients; *EDA*, Epidural Group; *CRO*, Controlled-release oxycodone Group.

## Discussion

Recently published data suggest that CPP might appear in around 20% of patients after thoracotomy when postoperative pain is managed aggressively [1]. These good results are based on postoperative analgesia via the epidural route. To date the “gold standard” for postoperative analgesia after thoracic surgery are regional techniques [1,3,4]. To our knowledge there are no prospective results available on the incidence of CPP after aggressive systemic pain therapy with controlled-release opioids compared to epidural analgesia. The objective of our prospective study was to assess the incidence and quality of CPP in patients undergoing elective thoracic surgery with postoperative epidural analgesia versus controlled-release oxycodone pain regimen over a 6-months period. We found no patient (zero percent) with a neuropathic pain 6 months postoperatively.

Steegers et al. determined definite neuropathic pain to be present in 23% of the patients with CPP [8] also by using the painDETECT questionnaire, the same tool for detecting neuropathic pain. Steegers et al. performed the study retrospectively and the patients were treated with posterolateral thoracotomy. Therefore data are not comparable to ours examined in anteroaxillary thoracotomy patients. Maguire et al. recently reported the prevalence of neuropathic pain between 35-83% after thoracotomy [5]. The authors could generate data of 600 patients with thoracotomy over 7 past years, but neither the procedure e.g. the anaesthetic management, nor the pain management was standardized. Moreover the surgical management was not standardized, different staff is described, various sizes of chest drains are reported [5]. Finally again, data are not comparable to our design as all patients in this study underwent a posterolateral thoracotomy. Guastella et al. demonstrated an incidence of neuropathic pain at 29% after lateral or posterolateral thoracotomy [9]. Data were assessed prospectively, but cannot be compared to our data as the presence of CPP was inclusion criteria for this study.

In our hospital pericostal sutures are common practice and our good results underline the atraumatic approach and closure of our surgeons. Cerfolio et al. stated that intracostal sutures decrease the pain after thoracotomy, but they present only mean pain scores after 2 weeks until three months postoperatively, but no incidences or percentages of patients who develop CPP [10]. All patients underwent a posterolateral thoracotomy with epidural analgesia in this retrospective study, we, therefore, cannot compare these results to ours. Our patients underwent an anteroaxillary surgical approach, the standard approach in our thoracic hospital. Little is known about the impact of the surgical approach on the incidence of CPP. Nasotti et al. compared a muscle sparing approach versus posterolateral thoracotomy for pulmonary lobectomy and found no difference in postoperative pain results and incidence of postthoracotomy pain syndrome [11]. Recently published data focus for the first time on the effect of the surgical access and wound closure on postoperative pain [12]. According to Sakakura et al. patients after posterolateral thoracotomy experienced more pain than anteroaxillary thoracotomy patients. These data cannot be compared to our data, as they fail to describe incidences of CPP and neuropathic component, but describe mean pain scores within the groups. However, our study was not designed to answer the question if the surgical approach influences patients`outcome with regard to chronic pain.

The painDETECT questionnaire is classified as validated tool for assessing neuropathic pain [8,13]. However, the painDETECT questionnaire might have limitations in detecting CPP. Moreover, functional exploration would have increased the impact of our data [14].

Our pilot data indicate that postoperative epidural analgesia might not be superior to systemic oral analgesia with regard to establishing CPP. More RCT`s with a greater number of patients and postoperative functional exploration are necessary to investigate if consequent systemic pain regimen and postoperative epidural analgesia are equally effective in preventing CPP.

### Limitations of the study

One limitation of our study is that the study was not randomized and double-blinded. The investigator (JL) might have been biased.

Another limitation is the different number of drop outs in both groups. Therefore, there might be a bias in the results and in interpreting the results.

## Conclusion

These pilot data indicate that epidural analgesia is not superior to sytemic postoperative analgesia with regard to preventing chronic or neuropathic pain after thoracotomy.

## Abbreviations

CRO: Controlled-release oxycodone; EDA: Epidural analgesia; NRS: Numerical rating scale; CPP: Chronic postthoracotomy pain; ASA: American society of anaesthesiologist; POD: Postoperative day.

## Competing interest

The authors declare that they have no competing interests.

## Authors’ contributions

SK was responsible for the study and drafted the manuscript. JL performed the clinical investigations. GW carried out the statistical analysis. MH designed the data base and data collection. SW supported JL in performing the clinical investigations and SK in drafting the manuscript. GS participated in the design of the study and its coordination. All authors read and approved the final manuscript.
